# Sclerotinia sclerotiorum Response to Long Exposure to Glucosinolate Hydrolysis Products by Transcriptomic Approach

**DOI:** 10.1128/spectrum.00180-21

**Published:** 2021-07-14

**Authors:** Pari Madloo, Margarita Lema, Maria Elena Cartea, Pilar Soengas

**Affiliations:** a Group of Genetics, Breeding and Biochemistry of Brassicas, Misión Biológica de Galicia, Spanish Council for Scientific Research (MBG-CSIC), Pontevedra, Spain; b Department of Functional Biology, School of Biology, Universidade de Santiago de Compostela, Santiago, Spain; Broad Institute

**Keywords:** plant-pathogen interaction, isothiocyanates, indoles, plant secondary metabolites, *Brassica*

## Abstract

White mold disease, caused by the necrotrophic fungus Sclerotinia sclerotiorum, affects *Brassica* crops. *Brassica* crops produce a broad array of compounds, such as glucosinolates, which contribute to the defense against pathogens. From their hydrolysis, several products arise that have antimicrobial activity (GHPs) whose toxicity is structure dependent. *S. sclerotiorum* may overcome the toxic effect of moderate GHP concentrations after prolonged exposure to their action. Our objective was to identify the molecular mechanism underlying *S. sclerotiorum* response to long exposure to two chemically diverse GHPs: aliphatic GHP allyl-isothiocyanate (AITC) and indole GHP indol-3-carbinol (I3C). We found that the transcriptomic response is dependent on the type of GHP and on their initial target, involving cell membranes in the case of AITC or DNA in the case of I3C. Response mechanisms include the reorganization of chromatin, mediated by histone chaperones *hip4* and *cia1*, ribosome synthesis controlled by the kinase-phosphatase pair *aps1-ppn1*, catabolism of proteins, ergosterol synthesis, and induction of detoxification systems. These mechanisms probably help *S. sclerotiorum* to grow and survive in an environment where GHPs are constantly produced by *Brassica* plants upon glucosinolate breakdown.

**IMPORTANCE** *Brassica* species, including important vegetable crops, such as cabbage, cauliflower, or broccoli, or oil crops, such as rapeseed, produce specific chemical compounds useful to protect them against pests and pathogens. One of the most destructive *Brassica* diseases in temperate areas around the world is Sclerotinia stem rot, caused by the fungus Sclerotinia sclerotiorum. This is a generalist pathogen that causes disease over more than 400 plant species, being a serious threat to economically important crops worldwide, including potato, bean, soybean, and sunflower, among many others. Understanding the mechanisms utilized by pathogens to overcome specific plant defensive compounds can be useful to increase plant resistance. Our study demonstrated that *Sclerotinia* shows different adaptation mechanisms, including detoxification systems, to grow and survive when plant protective compounds are present.

## INTRODUCTION

Sclerotinia sclerotiorum is a necrotrophic fungal pathogen that attacks at least 408 species of plants from 278 genera, belonging to 75 families (1). *S. sclerotiorum* produces the so-called *Sclerotinia* stem rot or white mold in *Brassica* crops, whose symptoms include the rotting of leaves, stems, and pods, which causes important economic losses, especially for oil crops (2).

*Brassica* plants can produce a broad array of compounds that contribute to the defense against *S. sclerotiorum*. This includes regulatory hormones such as jasmonic acid, salicylic acid, and ethylene as well as defensive compounds like glucosinolates (2). These are secondary metabolites present in the *Brassicaeae* family, derived from amino acids. Depending on the side chain of the amino acid, glucosinolates can be classified as aliphatic (derived from methionine, alanine, valine, leucine, and isoleucine), aromatic (derived from phenylalanine or tyrosine), and indolic (derived from tryptophan) (3). Myrosinase enzymes hydrolyze glucosinolates into several chemically diverse products (GHPs). Hydrolysis occurs upon tissue breakdown produced by the attack of herbivores or necrotrophic pathogens (4). Depending on the nature of the glucosinolate side chain and the physicochemical conditions of the medium, such as pH, availability of ferrous ions, and the level and activity of specific protein factors, such as epithio specifier one, glucosinolates can hydrolyze into isothiocyanates (ITCs), nitriles, thiocyanates, epithionitriles, oxazolidine-2-thiones, epithioalkanes, and indoles. GHPs show antimicrobial effects *in vitro* against a broad range of fungal and bacterial plant pathogens (4, 5). Among GHPs, ITCs are the most toxic ones, even at low concentrations (6).

Glucosinolates and their GHPs have a role in the defense against *S. sclerotiorum in planta* (7) and *in vitro* (5, 8). Fungal response is dependent on the GHP chemical structure (5). However, to date, mechanisms through which GHPs exert their toxicity and the ways fungi can overcome it have not been completely understood. Broadly speaking, ITCs can act directly by covalent binding/modification of certain nucleophile-containing proteins, referred to as a target, or indirectly by activating gene transcription (9). *S. sclerotiorum* can overcome toxic effects driven by sublethal concentrations of ITCs after a short exposure, basically by inducing antioxidant defenses and efflux pumpers (8), similar to other necrotrophic fungi, such as *Alternaria brassicicola* (10). This would allow the initial plant colonization by the fungus. However, as the disease progresses, more glucosinolates hydrolyze and *S. sclerotiorum* is exposed, during the infection period, to the GHP action. In addition, within a plant, there are glucosinolates of different chemical classes simultaneously hydrolyzing to different GHPs, whose toxicity is structure dependent (11). In this line, this study aimed at identifying the molecular mechanisms underlying *S. sclerotiorum* response to long exposure to two chemically diverse GHPs. Identifying response mechanisms will allow advancing our understanding of the interaction of *Brassica* plants with *S. sclerotiorum*.

## RESULTS

### Phenotypic effect of GHP treatments on *S. sclerotiorum*.

**FIG 1:**
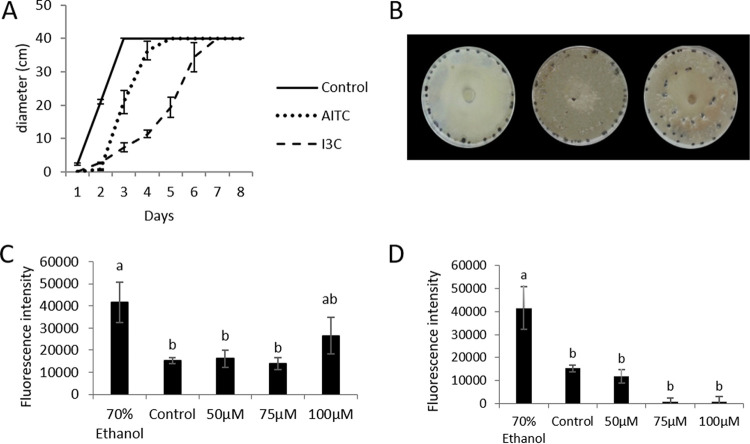
(A) Radial growth of Sclerotinia sclerotiorum in PDA medium with 100 μM allyl-isothiocyanate (AITC) or indol-3-carbinol (I3C) measured for eight consecutive days. (B) Production of sclerotia from Sclerotinia sclerotiorum grown in PDA medium under three different treatments (from left to right): control, AITC, and I3C. (C and D) Cell permeability assay of Sclerotinia sclerotiorum treated with AITC (C) and I3C (D) 40 min after the addition of SYTOX green. Letters at the top of the bars show separation of means by least significant differences at *P*  ≤  0.05.

*S. sclerotiorum* mycelia took more time to reach the end of the petri dish when they were treated with GHPs than control samples. This effect was clearer when samples were treated with I3C (Fig. 1A). Once AITC and control sample mycelia reached the end of petri dishes, they started producing sclerotia on the edge. However, sclerotia from I3C-treated samples also were produced in the midpoint of petri dishes (Fig. 1B). 100 μM AITC provoked membrane permeabilization, and it was half that by 70% ethanol (Fig. 1C). No permeabilization effect was observed for I3C (Fig. 1D).

### Transcriptomic analysis.

Averages of 14,960,380, 16,415,912, and 10,776,800 tag sequences were obtained from the control and samples treated with AITC and I3C. To offer a general result overview, GO analysis with all of the annotated transcripts was conducted for each comparison (GHP versus control and AITC versus I3C) with GO-Tool2. We describe the most important features found under the biological process (BP) category (see Table S1 in the supplemental material).

### AITC versus control.

Transcript analysis resulted in 212 significantly enriched GO terms (*P* ≤ 0.05 and −1 ≤ log_2_ fold change ≥ 1), from which 181 were induced and 31 were repressed. The most significant induced GO terms were related to mitosis and cell division (i.e., GO:1901989, GO:1901992, GO:1903047, and GO:0051301). Other categories were related to the organonitrogen compound metabolic process (GO:1901564) and the small-molecule metabolic process (GO:0044281). Otherwise, there is a large set of significantly enriched terms associated with the positive regulation of the proteasomal protein catabolic process (GO:1901800 and GO:0032436). The first six most significantly repressed GO terms are related to toxin biosynthetic pathways (GO:1901376, GO:1901378, GO:0045461, GO:0045460, GO:0009403, and GO:0009404). Several terms were related to apoptosis and cell death (GO:0006915, GO:0012501, GO:0008219, and GO:0016265). Other significant terms included processes related to mitochondrial transmembrane transport (GO:1990542 and GO:0045041), mitochondrial DNA repair (GO:0043504), and maturation of LSU-rRNA (GO:0000463 and GO:0000470).

### I3C versus control.

GO analysis of I3C versus control treatment rendered 197 significant GO terms, from which 190 were repressed and 7 were induced. Significantly induced GO terms were mostly related to amino acid synthesis, including serine (GO:0009070 and GO:0009092), threonine (GO:0009088), and sulfur amino acids (GO:0000097). The ubiquitin-dependent protein catabolic process via the multivesicular body sorting pathway (GO:0043162) and glyoxylate metabolic process (GO:0046487) were also significant. A large part of significant GO repressed terms were related to translation and ribosome biogenesis and assembly (GO:0002181, GO:0042273, and GO:0042255). Among the most significant GO downregulated terms, we could find several terms related to stress and stimulus response (GO:1901700, GO:0042221, GO:0006970, GO:0009408, and GO:0010035). Other significantly repressed terms were involved in the small-molecule metabolic process and nucleobase-containing small-molecule metabolic process (GO:0044281 and GO:0055086) and toxin biosynthetic process (GO:0009403).

### AITC versus I3C.

Expression of 296 GOs was higher in mycelia treated with AITC than in mycelia treated with I3C. Among them, we can find terms related to cell division, such as positive regulation of metaphase/anaphase transition of the cell cycle (GO:1902101), positive regulation of cell cycle phase transition (GO:1901989), or actin polymerization or depolymerization (GO:0008154). Other terms are related to catabolic processes, such as positive regulation of the proteasomal protein catabolic process (GO:1901800) and positive regulation of proteolysis (GO:0045862). Translation (GO:0006412), ribonucleoprotein complex assembly (GO:0022618), endoplasmic reticulum (ER)-nucleus signaling pathway (GO:0006984), cellular response to sterol depletion (GO:0071501), and fungus-type cell wall biogenesis (GO:0009272) were also upregulated by AITC. The expression of four GO terms was higher in the treatment with I3C compared to AITC: mitochondrial DNA repair (GO:0043504), cellular response to light stimulus (GO:0071482), mitochondrial DNA metabolic process (GO:0032042), and response to light stimulus (GO:0009416).

### Differentially expressed genes.

**FIG 2:**
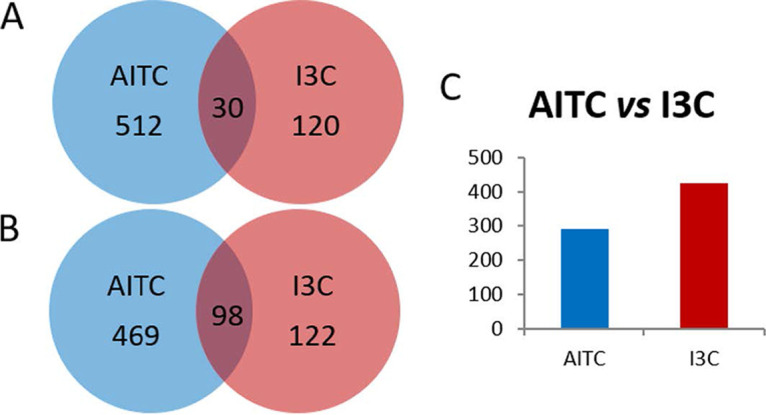
Venn graph showing overlapping up-DEGs (A) and down-DEGs (B) between the AITC versus control and I3C versus control comparisons. (C) Number of DEGs upregulated by AITC or I3C in the AITC versus I3C comparison. Identification of DEGs was performed based on a false discovery rate (FDR) of ≤0.05 and −1 ≤ log_2_ fold change (FC) ≥ 1.

**FIG 3:**
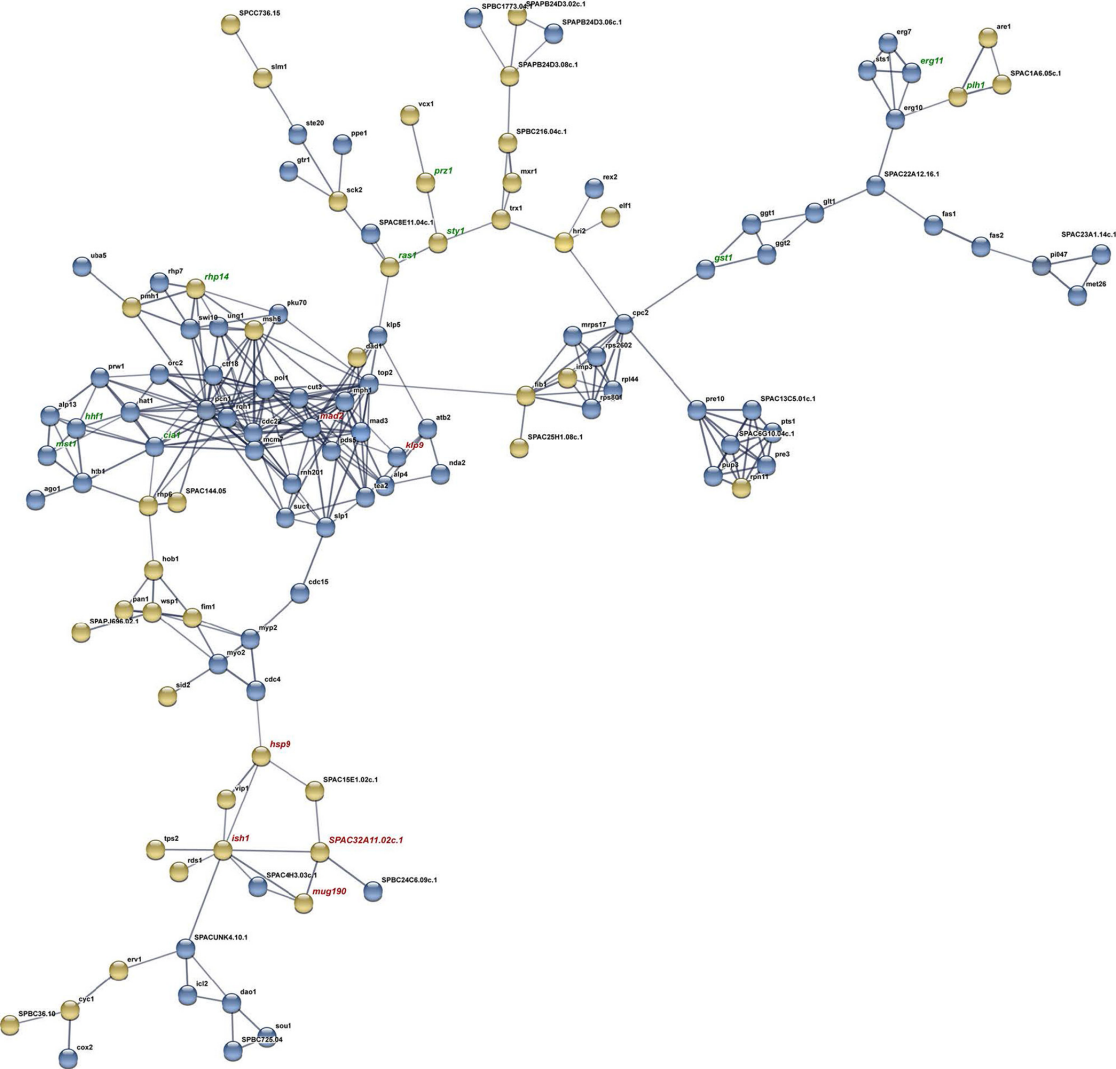
Protein association network built with all DEGs of the AITC versus control comparison. Line thickness indicates the strength of data support, which is based on experimental results and coexpression analysis in Schizosaccharomyces pombe. Blue and yellow nodes show DEG up- or downregulation. The names of DEGs cited throughout the text are written in green. The names of DEGs shared with the I3C versus control comparison are shown in red. Description of DEGs is given in Table S2.

**FIG 4:**
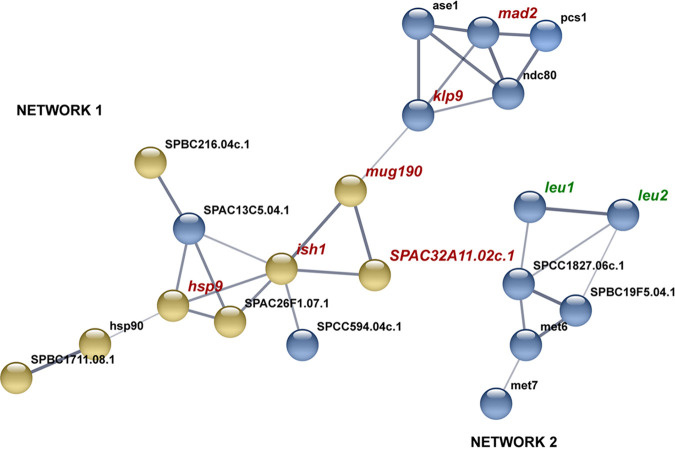
Protein association network built with all DEGs of the I3C versus control comparison. Line thickness shows the strength of data support, which is based on experimental results and coexpression analysis in Schizosaccharomyces pombe. Blue and yellow nodes indicate DEG up- or downregulation. The names of DEGs cited throughout the text are written in green. The names of DEGs shared with the I3C versus control comparison are shown in red. Description of DEGs is included in Table S2.

There was a higher number of DEGs in response to AITC than in response to I3C compared to control samples. Thirty genes were commonly induced by both treatments, and 98 were repressed (Fig. 2A and B). In the AITC versus I3C comparison, 291 DEGs were induced in response to AITC, and 427 were induced in response to I3C (fig. 2C). Relationships among DEGs from each comparison were deduced by using the web tool STRING (v10.5). One hundred twenty-three DEGs from the AITC versus control comparison interact in a single network (Fig. 3). DEGs interact in several clusters related to sterol and lipid synthesis, proteasome, translation, cell division, and DNA replication (Fig. 3, Table S2). Twenty-one DEGs interact in two different networks in the I3C versus control comparison (Fig. 4, Table S2). Part of network 1’s structure (including DEGs *mad2*, *klp9*, *mug190*, *ish1*, *hsp9*, and SPAC32A11) is shared with the network of the AITC versus control comparison. In both comparisons, *mad2* and *klp9* are induced and *mug190*, *ish1*, *hsp9*, and SPAC32A11 are repressed.

**FIG 5:**
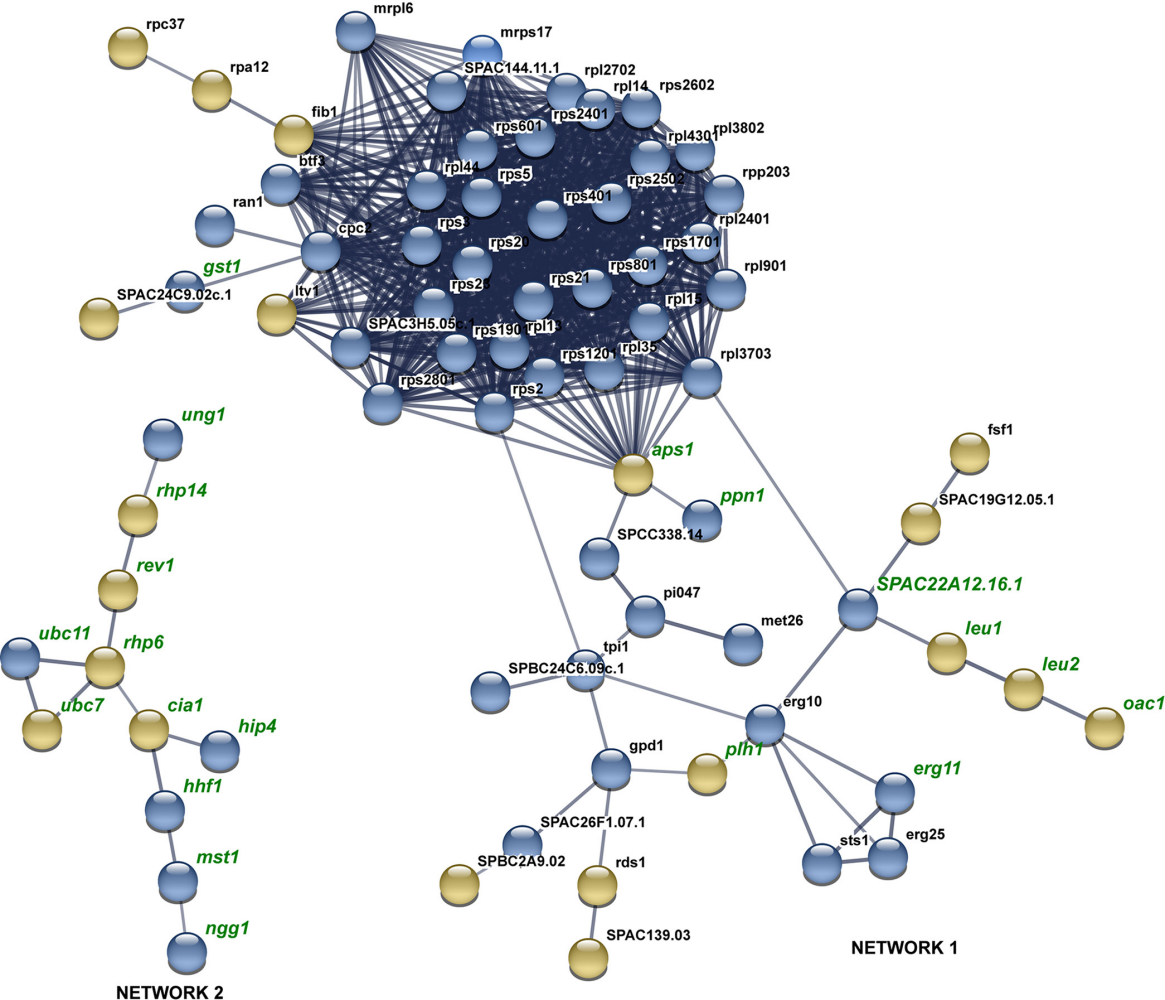
Protein association network built with all DEGs of the AITC versus I3C comparison. Line thickness shows the strength of data support, which is based on experimental results and coexpression analysis in Schizosaccharomyces pombe. Blue nodes show upregulated DEGs by AITC, and yellow nodes show upregulated DEGs by I3C. Description of DEGs is included in Table S2.

Seventy-four DEGs are interacting in the AITC versus I3C comparison (Fig. 5, Table S2) in two different networks. In network 1, there is a cluster of structural proteins related to ribosome synthesis, which are more highly induced by AITC than by I3C. All these structural proteins are related to protein Aps1, which is repressed by I3C and induced by AITC. In this network, a cluster of DEGs related to ergosterol synthesis (induced by AITC) and leucine synthesis (induced by I3C) also appears, interacting with protein SPAC22A12.16.1. In network 2, several DEGs, *hip4, hhf1, mst1*, and *ngg1*, are induced by AITC, whereas DEGs *rhp6, ubc7, rev1*, and *rhp14* are induced by I3C. Both branches are connected by DEG cia1, which is induced by I3C and repressed by AITC.

DEGs in the three comparisons that were related to detoxification of GHPs and toxin biosynthesis are shown in Table 1.

**TABLE 1:** List of DEGs related to detoxification of genotoxic substances and toxin biosynthesis^*a*^

Gene name	Description	AITC vs control		AITC vs I3C		I3C vs control	
		FDR	Log_2_	FDR	Log_2_	FDR	Log_2_
Detoxification							
BcDW1_206	Putative *mfs* transporter protein	0.042	1.038				
BcDW1_3316	Putative *mfs* multidrug transporter protein					0.009	2.510
BcDW1_10619	Putative *mfs* monocarboxylate transporter protein			0.025	−2.977	0.019	3.416
SPAC1399.02	Uncharacterized *mfs*-type transporter	0.004	1.335	0.000	1.279		
SS1G_12101	Putative *mfs* transporter protein					0.000	1.257
*cdr1*	Pleiotropic ABC efflux transporter of multiple drugs					0.038	1.216
SS1G_10295	Putative glutathione *S*-transferase	0.001	2.466	0.032	1.210		
SS1G_14440	Putative glutathione *S*-transferase	0.002	1.250			0.001	1.840
*gst1*	Glutathione *S*-transferase	0.000	1.343	0.000	1.341		
SS1G_12040	ITCase	0.000	4.667	0.000	3.087		
Toxin biosynthetic process							
SS1G_04352	Putative uncharacterized protein	0.000	−1.985			0.026	−1.002
SS1G_05105	Putative uncharacterized protein	0.000	−4.490	0.001	−2.288	0.000	−2.203
*stcC*	Putative sterigmatocystin biosynthesis peroxidase	0.004	−4.080				
*tcmN*	Multifunctional cyclase-dehydratase-3-O-methyl transferase			0.000	−3.530	0.000	2.798
*ccsA*	Polyketide synthase-nonribosomal peptide synthetase	0.000	1.656	0.000	2.230		

a Annotations of DEGs were performed via BLASTX to the UniProt database (Swiss-Prot and TrEMBL) and TrinityRNA-Seq. The false discovery rate (FDR) of each DEG and its log_2_ fold change are shown for each one of the comparisons where they are significant.

## DISCUSSION

### GHPs alter fungal growth and cell cycle.

Treatment with both GHPs produced slower mycelium growth than control conditions. This effect was more evident in fungi treated with I3C than with AITC. Moreover, in I3C-treated samples, the number of transcripts found was lower than that in AITC samples or the control group, indicating that, at the same concentrations, I3C is more toxic than AITC on *S. sclerotiorum*.

Several common transcripts in both treatments have an antiapoptotic function, of which only *casA* and *crz1* were significantly repressed by both GHPs and *ppk31* was significantly repressed by AITC. *casA* is a metacaspase-1, which is a positive regulator of ER stress-induced cell death (12) in other fungi (Aspergillus nidulans and A. fumigatus). *crz1* is a target of the calcineurin protein that is related to resistance to antifungal molecules and homeostasis of cations in Saccharomyces cerevisiae (13).

*ppk31* is involved in the induction of autophagy and life span regulation. *ppk31* increases the chronological life span of the S. pombe DY8531 strain (14). A life span can be modified by changing the number of mitotic divisions that a cell can undergo during its lifetime or by modifying the time a cell remains viable. In the first case, the term employed is replicative life span; in the second case, it is called chronological life span (14). Therefore, by inhibiting *ppk31*, fungi treated by AITC may stop the chronological life span to increase the replicative life span.

Unlike AITC, no GOs associated with mitosis were found in mycelia treated with I3C. Chronological life span in S. cerevisiae is related to amino acid availability. The addition of isoleucine, valine, and leucine extended the average chronological life span in S. cerevisiae with the limitation of nitrogen and carbon sources (15). Accordingly, in the AITC versus I3C comparison, DEGs *leu1* and *leu2*, key genes in leucine synthesis, were induced by I3C. Furthermore, these DEGs coexpressed with *oac1*. *oac1* exchanges isopropylmalate by oxalacetate from mitochondria to cytosol. Leucine synthesis is related to this transporter, since its precursor pyruvate can be obtained by oxaloacetate, once it is in the mitochondria (16). Tsang et al. (17) proved that *oac1* is related to life span extension in S. cerevisiae through NAD^+^ regulation. In the GO analysis, the glyoxylate cycle was upregulated by I3C compared to control samples. When simple sugars are scarce, lower eukaryotes employ the glyoxylate cycle to obtain energy from two-carbon compounds (18). There is a link between the glyoxylate cycle and chronological life span in S. cerevisiae. *icl1* is a key gene in the glyoxylate cycle and was induced by I3C. Deletion of this gene in S. cerevisiae under calorie restriction provokes a decrease in chronological life span (19). Following reference 19, the chronological life span is favored by glyoxylate/gluconeogenesis flux, contrary to the ethanol and acetate flux via the Krebs cycle and mitochondrial respiration. One of the responses of fungus treated with I3C may be to increase the chronological life span of mycelium cells. Therefore, it seems that fungi are following two different growth strategies to cope with the toxic effects of both GHPs.

Both GHPs provoke a delay in mycelium growth. However, once fungi treated with AITC start growing, the shape of the graph of growth over time is like that of control samples. However, I3C-treated samples show a different growth pattern. The differential expression on genes related to the cell cycle may underlie differences in growth patterns.

There are several transcripts related to the cell cycle that were commonly regulated in AITC versus control and I3C versus control comparisons. DEGs *mad2* and *klp9* were induced by both ITCs. They are related to spindle dynamics during mitosis (20, 21). *ish1* and *hsp9* are part of the mitogen-activated protein kinase (MAPK) pathway, and both are downregulated by both GHPs. The expression of *ish1* increases after S. pombe exposure to osmotic stress or glucose deprivation (22). The function of *hsp9* is related to the regulation of the cell cycle by increasing the length of S. pombe cells and decreasing their growth rate (23).

Other common DEGs were regulated differentially by both treatments in the AITC versus I3C comparison. DEGs from network 2 are mostly related to histone modification and DNA repair. Synthesis of histone H4 (DEG *hhf1*) is induced by AITC. Histone chaperones modulate nucleosome assembly and, therefore, the structure of chromatin (24). *hip4* and *cia1* in S. pombe (homologous to *hpc2* and *asf1* of S. cerevisiae) are chaperones acting on histones H3 and H4. *hip4* is a component of the HIR complex. Deletion of *hip4* provokes cycle delay (25). This gene is necessary for correct chromosome segregation. *cia1* is a repressor of the HIR-dependent histone genes. Mutants in *cia1* cause double-strand breaks in DNA, increase DNA sensitivity to nuclease, and subsequently activate the DNA damage checkpoint pathway. Consequently, life span is severely reduced (25, 26). AITC induces *hip4*, and I3C induces *cia1*.

The function of histone chaperones can be regulated by posttranscriptional modifications, leading to the regulation of various processes involving DNA, such as transcription, reparation, or condensation (27). DNA replication is regulated by the acetylation of histones H3 and H4 (28). This function is carried out by DEGs *ngg1* and *mst1* (29) when fungi are treated with AITC. On the other hand, ubiquitination of DNA is induced by I3C, via *rhp6* and *rhp14*. Both DEGs, together with the DNA polymerase *rev1*, act in DNA repair (30–32), contributing to genome stability.

Therefore, regulation of chromatin structure is dependent on GHPs. In the case of AITC-treated samples, this reorganization seems to be involved in mitosis and DNA replication, where, in the case of I3C, it may be related to DNA repair. Differences in growth performance between both samples may be due to DNA reparation, being a possible direct or indirect target of I3C.

### GHPs alter protein and lipid metabolism.

Translation and ribosome synthesis were induced by AITC and repressed by I3C. Since mitosis of AITC treated cells is induced, they need to produce new proteins. However, in the case of I3C cells, with slower growth and the need to repair their DNA, ribosome synthesis is a highly energy-consuming process. Eukaryotic cells cease the production of ribosomes very rapidly upon unfavorable conditions to ensure survival (33). In the network constructed with DEGs of the AITC versus I3C comparison, a big cluster of ribosome-associated proteins is induced by AITC. All these proteins are linked to *asp1*, which is induced by I3C.

The inositol pyrophosphates (PP-InsPs) play a central role in signaling and reprogramming cell metabolism through their phosphorylation/dephosphorylation. Asp1 is an inositol kinase that participates in the synthesis of the diphosphate group of PP-InsP5 and (PP)2-InsP4 (34), and previous authors (34) found that a considerable amount of proteins isolated from S. cerevisiae that interact with 5PP-InsP5 are related to ribosome biogenesis and maturation. *asp1* coexpressed with *ppn1*, which is an endopolyphosphatase, and catalyzes the hydrolysis of polyphosphate chains. This last DEG was induced by AITC. Therefore, it seems that the system *aps1-ppn1* is controlling ribosome synthesis in fungi treated with GHPs.

Both treatments induced protein catabolism. AITC induced the ubiquitin-proteasomal system, whereas I3C induced the multivesicular (MVB) body sorting pathway. The ubiquitin-proteasome system has an essential role in cell growth and differentiation, survival, stress tolerance, and adaptation to environmental changes (9, 35). This mechanism is critical for cell survival (36). Formation of MVB takes place by invagination of the endosome membrane. MVB is degraded by fusing to lysosomes (35). Both mechanisms serve to regulate protein turnover and degrade the damaged ones. Proteins to be degraded by both systems are joined to ubiquitin as a signal (35). In the AITC versus I3C comparison, two ubiquitination-related DEGs were significantly expressed. *ubc11* was induced by AITC, whereas *ubc7* was induced by I3C.

Catabolism of proteins may be associated with mitosis and damage caused by GHP treatments. Secretory proteins are primarily assembled and folded in the ER. An increment in misfolded proteins inside ER lumen may be provoked by an increment in the protein load, but it can also respond to oxidative stress and modifications in lipids and calcium (37). Lipid perturbation seems to happen in cells treated with AITC, because this treatment diminished the permeabilization of *S. sclerotiorum* cells, whereas I3C did not affect it. The sterol biosynthetic process was induced in fungi treated with AITC compared to I3C. Sterols play an important structural role in regulating membrane fluidity and permeability (38). Besides, DEG *wA* was induced by AITC while repressed by I3C. This gene is related to melanin synthesis in fungi, which gives strength and rigidity to the cell wall. Consequently, it is related to cell permeability and turgor (39). In the AITC versus I3C comparison, DEG *plh1* was induced by I3C. This DEG codes for a phospholipid, diacylglycerol acyltransferase, related to triacylglycerol synthesis (40). Fungi treated with AITC probably modify the composition of cellular membranes by increasing the level of sterols compared to control samples and I3C treatments and decreasing triacylglycerols to increase their stability. Similarly, the ergosterol synthesis pathway and, specifically, gene *erg11* is a target of azole and non-azole fungicides in different fungal species (10, 41, 42).

### GHPs induce detoxification systems and repress toxin biosynthesis.

As a detoxification mechanism, glutathione *S*-transferases were induced by *S. sclerotiorum* (8) and *A. brassicicola* (4, 43) after exposure to several ITCs. In line with this, several glutathione transferases were induced by both treatments; coincidentally, SS1G_10295 was also induced after treatment of *S. sclerotiorum* with AITC (8). Exposure to AITCs causes oxidative damage in fungi (4, 8, 10). Conjugation of glutathione with GHPs may cause a drop in free glutathione and the inhibition of glutathione reductase. Hog1 is a MAP kinase related to the regulation of the oxidative stress response and was induced after exposure of *A. brassicicola* to AITC (4, 44). However, in this work, *sty1* (homologous to *hog1*) was repressed after AITC treatment, probably because, when samples were taken, oxidative stress caused by AITC was reduced after several days of adaptation.

Induction of MFS and ABC transporters is a common defense mechanism to detoxify fungus cells (10, 42, 45). In this case, several MFS and one ABC transporter were induced by GHP treatments. Induced transporters were not coincident between treatments, indicating the substrate specificity. MFS transporter SS1G_12101 was also induced in *S. sclerotiorum* after the invasion of *B. napus* plants (46). SS1G_12040 was induced by AITC. Following reference 47, it is a *saxA* gene, responsible for the hydrolysis of ITCs to amines and allowing *S. sclerotiorum* to tolerate ITCs in the medium.

In GO analysis, the toxin biosynthetic process was downregulated by both GHPs. This GO term includes genes that are similar to sterigmatocystin coding genes. Sterigmatocystin is a polyketide compound produced by several Aspergillus species and is a precursor of aflatoxin. Repression of toxin-related genes by fungi in the presence of fungicides has been previously described (42) and may respond to a need to derive energy to prime metabolism.

### Conclusions.

Moderate concentrations of GHPs inhibit the growth of *S. sclerotiorum in vitro*. However, after long exposure, fungi may overcome their toxic effects and grow at the same level as nontreated fungi. We give an overview of the adaptation mechanisms of *S. sclerotiorum* to two chemically diverse GHPs, the aliphatic AITC and the indole I3C. We show that adaptation mechanisms are dependent on the type of GHP and on their initial target, involving cell membranes in the case of AITC or DNA in the case of I3C. Adaptation mechanisms include the reorganization of chromatin, mediated by histone chaperones *hip4* and *cia1*, ribosome synthesis controlled by the kinase-phosphatase pair *aps1-ppn1*, catabolism of proteins, ergosterol synthesis, and the induction of detoxification systems. These mechanisms probably help *S. sclerotiorum* to grow and survive in an environment where GHPs are being produced by *Brassica* plants upon glucosinolate breakdown.

## MATERIALS AND METHODS

### Fungal growth and treatment with GHPs.

We collected *S. sclerotiorum* isolate MBG-*Ss*2, employed in the experiments on *B. napus* in Spain, and it is currently maintained at MBG-CSIC. To provide fresh colonies for the experiments, surface-sterilized sclerotia were cultivated on potato dextrose agar (PDA; Difco) plates and incubated at 24°C in the dark for 4 days. Fresh colonies were obtained through routine growth of mycelium-agar plugs from the margin of the fungal colony on PDA and incubated at 24°C for 72 h. GHPs were purchased from Sigma-Aldrich, including allyl-ITC (AITC) (36682) and indole-3-carbinol (I3C) (I7256-5G). Fungal growth in 2× YPG medium was monitored spectrophotometrically for 4 days at 25°C with the addition of each GHP in concentrations ranging from 0 to 250 μM. A sublethal concentration of 100 μM for both compounds then was selected to be used in the transcriptomic analysis.

Fungi were grown in PDA medium supplemented with each GHP, which was added to the medium before solidification to reach a concentration of 100 μM. Petri dishes were coated with one layer of cellophane membrane (catalog no. 165–0963; Bio-Rad). Agar plugs of 2.5-mm diameter containing fresh fungal colonies then were transferred to petri dishes and incubated at 24°C in darkness. After 5 days, mycelium mass was harvested from cellophane membranes and homogenized in liquid nitrogen. The resulting material was immediately used to isolate total RNAs. Two replicates per treatment and control sample were carried out.

In another experiment, the diameter of fungal mycelium growing radially from the center was measured for 8 days, until it reached the end of petri dishes.

### RNA isolation, library preparation, and sequencing.

RNA was extracted with a Spectrum plant total RNA kit (Sigma, MO, USA) according to the manufacturer’s protocol. In order to remove any traces of genomic DNA, the RNA was treated with DNase by following the manufacturer’s instructions. Massive analysis of 3′-cDNA end (MACE) libraries was generated by using GenXPro’s MACE kit (GenXPro GmbH, Frankfurt, Germany) as described previously (48). Briefly, cDNA from 2 μg of total RNA was randomly fragmented, and biotinylated 3′ ends were captured after binding to a streptavidin matrix. A library ready for high-throughput sequencing was prepared by using TrueQuant adapters included in the kit. The library consisted of 50- to 700-bp-long fragments derived from the 3′ end from cDNAs. The 5′ ends from the libraries were sequenced by using an Illumina HiSeq 2000 version 4 chemistry (Illumina, Inc., San Diego, CA, USA).

NextSeq-derived sequence reads were processed with GenXPro’s online in-house MACE analysis pipeline. Briefly, libraries were sorted according to their respective index, and all duplicate reads detected by the TrueQuant technology were removed from raw data sets. Additionally, quality filtering (by software Cutadapt [https://github.com/marcelm/cutadapt/]) and poly(A) tails were clipped by an in-house python script. Reads were aligned to reference sequences using bowtie2. On this genome, since the functional annotation was poor, a cluster_bed step was performed. Reads that successfully map the genomic regions with no annotation (feature) were clustered. Clusters (consensus sequences) were annotated via BLASTX to the UniProt database (Swiss-Prot and TrEMBL). Reads that did not match the genome were taken into a *de novo* assembly step and annotated via BLASTX to the UniProt database (Swiss-Prot and TrEMBL). The assembly was completed with Trinity RNA-Seq (https://github.com/trinityrnaseq/trinityrnaseq/wiki). As for the functional annotation, UniProt (http://www.uniprot.org/) and NCBI fungal sequences were employed.

### Bioinformatic analysis.

In order to have a general result overview, Gene Ontology (GO) analysis with all the annotated transcripts was done for each comparison (GHP versus control and AITC versus I3C) with GO-Tool2. The likelihood of a specific GO term was calculated by using Fisher’s exact test.

Those transcripts with a false discovery rate (FDR) of ≤0.05 and −1 ≤ log_2_ fold change (FC) ≥ 1 were considered to be differentially expressed genes (DEGs) between treatments. The web tool STRING (v. 10.5) was used to study the interconnections between DEGs. In addition to well-supported protein–protein interactions experimentally observed, this resource includes indirect and predicted interactions on top (49). However, there is little information about the interactions among proteins in the *S. sclerotinia* genome. Relationships among proteins were deduced by employing the genome of Schizosaccharomyces pombe as a reference. Ten percent of annotations were found, and there is much more experimental evidence of interactions among proteins than in *S. sclerotiorum*.

### Determination of membrane permeabilizing potential in *S. sclerotiorum* treated with GHPs.

The SYTOX green uptake assay was carried out by following reference 50. When plasma membrane integrity is compromised, the influx of the dye and binding to DNA causes a significant increase in fluorescence. In brief, 10 μl of fungal mycelium suspension (optical density [OD], 0.37) was added to 100 μl of 2× YPG liquid medium (pH 4.5) and incubated for 48 h at 27°C. AITC or I3C then was added to get the final concentrations of 50, 75, and 100 μM; 0.2 μM SYTOX green was added to each sample. Samples showing 100% permeabilization were prepared by degrading cells with 70% ethanol. Fluorescence reads were carried out over a time course of *t*  =  0 to *t*  = 210  min using a Promega fluorescence measurement system (GloMax) at an excitation wavelength of 480 nm and an emission wavelength of 530 nm. Fluorescence values were corrected by the absorbance of samples measured at 600 nm. Measurements were carried out in triplicate.

### Data availability.

Data supporting the findings of this study are openly available in the Gene Expression Omnibus repository under reference number GSE144344.
